# The oral repellent – science fiction or common sense? Insects, vector-borne diseases, failing strategies, and a bold proposition

**DOI:** 10.1186/s40794-023-00195-9

**Published:** 2023-06-28

**Authors:** Irmgard L. Bauer

**Affiliations:** College of Healthcare Sciences, Academy - Tropical Health and Medicine, James Cook University, Townsville, QLD 4811 Australia

**Keywords:** Systemic repellent, Topical repellents, Spatial repellents, Mosquitos, Chemo-sensation, Neglected tropical diseases, Salt fortification, Insect decline, Biodiversity

## Abstract

Over the last decades, unimaginable amounts of money have gone into research and development of vector control measures, repellents, treatment, and vaccines for vector borne diseases. Technological progress and scientific breakthroughs allowed for ever more sophisticated and futuristic strategies. Yet, each year, millions of people still die or suffer from potentially serious consequences of malaria or dengue to more recent infections, such as zika or chikungunya, or of debilitating consequences of neglected tropical diseases. This does not seem value for money. In addition, all current vector control strategies and personal protection methods have shortcomings, some serious, that are either destructive to non-target species or unsatisfactory in their effectiveness. On the other hand, the rapid decline in insect populations and their predators reflects decades-long aggressive and indiscriminate vector control. This major disruption of biodiversity has an impact on human life not anticipated by the well-meaning killing of invertebrates. The objective of this paper is to re-examine current control methods, their effectiveness, their impact on biodiversity, human and animal health, and to call for scientific courage in the pursuit of fresh ideas. This paper brings together topics that are usually presented in isolation, thereby missing important links that offer potential solutions to long-standing problems in global health. First, it serves as a reminder of the importance of insects to human life and discusses the few that play a role in transmitting disease. Next, it examines critically the many currently employed vector control strategies and personal protection methods. Finally, based on new insights into insect chemo-sensation and attractants, this perspective makes a case for revisiting a previously abandoned idea, the oral repellent, and its use via currently successful methods of mass-application. The call is out for focused research to provide a powerful tool for public health, tropical medicine, and travel medicine.

## Introduction

Since time immemorial, insects pestered humans and brought disease, potentially devastating communities [1]. In 2020, there were 241 million malaria cases and 627,000 deaths worldwide [2]. An estimated 100–400 million dengue infections occur each year; about half the world’s population is now at risk [3]. Almost 900 million people remain threatened by lymphatic filariasis and require preventive chemotherapy [4]. An estimated 700,000 to 1 million new cases of leishmaniasis occur annually [5]. Many of the twenty neglected tropical diseases (NTDs) are vector-borne [6], recent insights include Buruli Ulcers [7], and still require more effort in prevention and control [8, 9]. Climate-driven expansion of, for example, *Aedes*-habitat into temperate regions will pose a considerable risk to naïve and unprepared populations with the potential of rampant outbreaks [10].

The economic burden caused by malaria [11], or dengue [12, 13] is considerable; stigma, discrimination and disability through NTDs add to the cost [14]. Vector-borne infections are also of concern to people exposed short-term, such as scientists, missionaries, expatriates, or military personnel. The risk to travellers is much reported [15, 16] and forms the core of travel medicine. Apart from carrying diseases, insects can be an arduous nuisance as camping in Finland or barbequing in Australian backyards attest. However, insects are a significant part of the food web, even if eradicating them sounds appealing [17].

Insects have a poor image. People do not like insects; most research goes into exterminating them. However, historical evidence confirms the often-fruitless fight against their existence. Unimaginable amounts of money have gone into decades of research and development into vector control, personal protection, treatment, and vaccines. Isolated victories do not deal with the core problem. While we celebrate ever more fantastic ground-breaking strategies made possible by modern biotechnology, millions of people still die or suffer from debilitating illnesses. This does not seem value for money.

The catalyst for this paper was research on tourists’ use of repellents in the Amazon region [18] (Table 1).

**Table 1:** Repellents - personal observations and notes

To understand the context of research on tourists’ (*n* = 373) use of personal protection from insect bites, I accompanied as paying client one group each of the two participating operators (Group 1: 7 clients, all-camping, 7 days/6 nights; Group 2: 9 clients, camping/lodge, 9 days/8 nights) on trips to Manu National Park in the Peruvian Amazon. I observed their protective measures throughout the day and on night excursions, and at various distances from potential insect breeding and resting sites or areas of congregation, such as insect clouds hovering a few centimetres above the river to await the emerging swimmer.
Both operators had briefed their clients thoroughly prior to the trip and emphasised the need for protection from insects. Therefore, it came as no surprise that all of them had brought plenty of repellent from home, and that some used it often and generously. The DEET content varied considerably; some were slow-release preparations. However, everybody got bitten, some very badly with multiple angry red lumps. Toilet-breaks revealed a previously neglected area of application: women’s buttocks. Tourists commented ‘I had zillions of bites’, ‘undeterred by DEET’, ‘attacked very badly’.
Believing that people might be less compliant than assumed led to me demonstrate to myself and others that consequent, correct and frequent use of repellent does prevent bites. During the two weeks in the jungle, I used a variety of repellents, lotions, or sprays, many a commercial sample provided at travel medicine conferences, including repellents for military use, and appropriate clothing (often 2 layers). My efforts worked well during the first day while not quite yet in the rainforest. However, from day two, insects bit through repellent - at times even through freshly applied product. The identity of the biting insects (mosquito, sand fly, midges) could not be established but did not matter at the time; the nuisance was obvious, and any infected insects could not be identified anyway. For two days, one 100% DEET preparation was used, which only worked for 1 h rather than the suggested 10 h. In short, despite correct use, the repellents did not repel. Out of curiosity, I applied the juice of the traditionally used Huito fruit (*Genipa americana* L.) in two large patches on one arm. Once the skin adopted the typical dark-blue stain, lasting for several days, the patches remained bite-free.
Humidity, sweat, heat and abrasion will all have influenced the efficacy of the various repellents, and the observed sample was small. But this trip happened in real life! While some products may have performed beautifully in a laboratory, they did not protect people on location.
The outcome for those who used repellents was disappointing. Reasons of those who did not use repellents correctly were: thinking they can judge the situation and need for repellents, thinking there were no insects until too late, it was too humid and too hot, there was no water to wash hands, not knowing about potential diseases or that they would get them. Others forgot, could not be bothered, or sprays stung in eyes and nose, especially when applied in windy conditions or on boats on the river.
The use of personal insect repellents favoured us who had money and access to the products. Local tourism workers, such as boatmen or cooks, did not have repellents. Pathogen-carrying vectors do not distinguish between foreign tourists and local residents. We use repellents for a short time to protect us from diseases that affect millions of people in poorer countries. Cost, unavailability, or impracticality of use, make the advice to people in endemic regions quite pointless. Repellents are hardly a reliable way to lower the burden of global vector-borne diseases. What would do a better job?

Having previously applied repellent obediently, vector-borne disease prevention now raised many questions. This paper will re-visit an idea from the 1960s, pursued by the US military, then seemingly abandoned, and re-assess it from a contemporary perspective based on current knowledge in relevant fields.

Most papers on vector control focus on, and measure success in, the defeat of the menace. The argument presented in this paper starts by giving the insects the right to reply. To do so, it is necessary to remind ourselves of insects’ roles and purpose, before examining current control measures. Finally, to counteract the currently unsatisfactory protection from vector-borne diseases, and to take advantage of new knowledge and technology, this paper builds a case for renewed research and development efforts into oral repellents.

## Insects – the largest biomass on earth

An estimated three quarters of all species in the Kingdom Animalia are insects (Phylum Arthropoda, Class Insecta). With perhaps as many as 1 million described, there may be up to 6.5 million yet undetected or undescribed and, therefore, unclassified [19]. These figures vary widely and are all speculative [20]. The first hexapod fossils date from the Devonian Era (400 million years ago), while more evolved insects date back to the Cretaceous time (135–65 million years ago) [19]. The absence of fossils during long periods suggests insect mass extinctions, which leave early taxonomic gaps unfilled. The taxonomy of modern insects is exquisitely complex, and discovery of new species as well as better methods of identification require periodical re-classification and taxonomy re-shuffles. In terms of biogeography, one finds the highest biodiversity in rainforests along the equator whereas the abundance of fewer species increases towards the poles. Changing temperatures and habitats influence distribution and abundance [19]. Insects are primarily terrestrial but also inhabit water except for deep oceans [21]. They were the first animals to fly, giving them the advantage to find food and better living conditions, and to escape predators [21]. Their exoskeleton gives them strong protection from the environment and an advantage over humans and other Chordata with their fleshy, vulnerable parts exposed on the outside.

### The rise of entomology

Records from China and Japan indicate that, in antiquity, humans were familiar with insects as useful providers, but also as pests of body and crops, and with forms of pest control [22]. In ancient Middle East, Egypt revered hornets as symbols of power, flies represented courage; the sacred scarab (*Scarabaeus sacer*) played a role in medicine, divination, and sorcery. From Mesopotamia emerged a grouped taxonomy, and in biblical Israel, King Solomon is said to have left a good description of insect behaviour [23]. Again, insects were important as pests. Plagues represented divine punishment; nevertheless, people employed physical, chemical, and biological control measures. Of the ten plagues in the Bible, insects (lice, flies, locusts) caused three [23].

In ancient Greece, Homer (c850BC) mentions insects, but most influential for the next 1500 years was the classical biology of Aristotle (384-322BC) with the first systematisation of insects based on knowledge and assumptions. Ancient Rome neglected science in favour of practical application and studies of agricultural pests. Plinius maior’s (c23-79AD) eleventh book of *Naturalis Historia* [24] compiles collections about insects in art, jewellery, behaviour and control but without original research [25]. In medieval times, scientific research was curtailed severely by prevailing mysticism and dogmatism, though some monasteries preserved classical works. In Spain, contact with Arabian science and translations of leading manuscripts added to current knowledge. In the New World, Aztecs prepared scarlet red from scale insects. Pre-Columbian pottery depicts lice, sandfleas, and outcomes of vector-borne disease, such as leishmaniasis. Similar finds point to insect knowledge and use in Africa [25].

From the Renaissance to the 17^th^ century, the restriction to religion-oriented thought limited original inquiry, which led to extraordinary situations. Pests, such as locusts, were tried in secular and ecclesiastical courts, represented by defence counsels. Persistence of plagues was threatened with excommunication. Failing that, some specimens were brought before court and executed [26]. Since the Church only recognised Aristotle and Plinius, absurd and imperfect depictions and descriptions of insects (and their use in medicine) appeared in theologically oriented literature based on fanciful assumptions of nature [26]. However, there were exceptions. Ulisse Aldrovandi (1522–1605), relying on pure nature research, was the first to establish systematic entomology as a science (*De Animalibus Insectis, 1602*). From then on, similar works appeared all over Europe, facilitated by increasing inventions and discoveries. With the arrival of the microscope, William Harvey’s (1578–1657) discovery of blood circulation not only in animals but also in insects, and Francisco Redi’s (1626–1698) demonstration that insects emerged from eggs rather than, as was assumed, from dirt and decay, facilitated an abundance of research. Three famous anatomists, Marcello Malpighi (1628–1694) – detail, Jan Swammerdam (1637–1685) – metamorphosis, and Anthonie van Leeuwenhoek (1632–1723) – improved microscopy (see his observation of a bee [27]), prepared the ground for artists to produce exquisitely precise illustrations of insects still in use today [26]. With evermore refined taxonomies by Linnaeus (1707–1778) and Fabricius (1745–1808) [28] and systemic specialisations in the 19^th^ and 20^th^ centuries [29], we arrive at the entomology of today. Now that we know so much about them, insects seem to be disappearing again, unexplained by diapause, the natural interruption of developmental progression in times of unsuitable environmental conditions [20].

Over the years, car drivers will have noticed after long road trips that hardly any dead insects need to be removed from windscreen and bonnet (‘windscreen phenomenon’) [30]. More specifically, a recent study demonstrated a 76–82% decrease in total insect biomass, not just vulnerable species, over the last 27 years. Of particular concern is that all traps were placed in 63 protected areas in Germany supposed to preserve intact ecosystems [31]. In Puerto Rican rainforests, over 36 years, ground-dwelling insects declined by 98%, canopy-dwelling species by 78% [32]. Acknowledged reasons are change in global temperatures, the use of pesticides, insecticides and herbicides, deforestation, modification of environments, intensive agriculture, and invasive species [30] as well as artificial lighting [33, 34]. While the extinction of wild mammals or birds is rightly lamented, the more dramatic decline in insects has taken place without much fanfare [35]. This is of grave concern considering the importance of insects for life on earth. In Sir David Attenborough’s words: ‘If we and the rest of the back-boned animals were to disappear overnight, the rest of the world would get on pretty well. But if the invertebrates were to disappear, the land’s ecosystems would collapse’ [36]. Scientific publications for general audiences (e.g., [37]) facilitate the creation of a ‘bioliterate society’ [38]. Role-playing, board and computer games, successfully employed in some countries, can teach the general public, children, farmers and stakeholders, especially in resource-poor regions, insect biology, vector control and crop management [39].

## Our dependence on insects

It is long established that ‘insects create the biological foundation for all terrestrial ecosystems’ ([40], p.10). Insects’ enormous scope of usefulness is awe-inspiring; yet, many of their roles are rarely contemplated [17]:

### Providing role

For tens of thousands of years, bees have provided humans with honey and wax [41], as rock art in Southern Spain from approx. 7000BC depicts. Over time, bees left the Old World, and apiculture became an important source of food and income for peoples around the world [42]. The cultivation of the silk moth (*Bombyx mori*) began in China around 4700BC [22, 43]. Scale insects produce shellac, a resin excreted by the female *Kerria lacca*, and cochineal (carmine red) stems from *Dactylopius coccus* living on prickly pears. Selected species find their way into jewellery [40]. Of increasing importance is insects’ role as a food source (entomophagy) for humans, livestock, wildlife, and insectivorous plant [17]. While western culture is still reluctant to eat insects, even if disguised, grasshoppers, crickets, grubs, spiders, and bugs improve the nutrition of millions of people [17, 44].

### Supporting role

Their ability to fly makes insects the main pollinator, not only for plants in general, but they are indispensable for crop pollination, hence crucial for food production [45]. This service is now well acknowledged, especially because shape and size of many flowers allow access only to a certain type or size of insect, making hand-pollination impossible [17, 19]. For example, several species visit cocoa flowers, but only tiny ceratopogonic midges can pollinate [46]. Unfortunately, these midges are highly vulnerable to habitat changes. For humans, this means: no midges – no chocolate. Insects may also play a role in seed dispersal [19]. Many species, e.g., termites or dung beetles, are responsible for the decomposition of plant and animal detritus, which leads to soil improvement and better water filtration [17, 19]. Twenty years ago, the services of beneficial wild insects in the US alone were valued at approx. US$ 60 billion annually [47].

### Regulating role

The presence or absence of insects serve as an indicator for habitat wellbeing. Insects deliver valuable services in the biological control of pests. Insect predators kill larvae or adult insects, e.g., dragonfly nymphs kill mosquito larvae [48], and lacewings control aphids and other pests [49]. Others kill through parasitism or parasitoidism where eggs are laid into or on the body of other insects, which serve as food for the developing larvae [19, 20]. The preservation of these biological control services is of utmost importance to human health as ‘unmanipulated ecosystems regulate abundances of pathogen and vector species through various food web interactions and habitat conditions’ ([17], p.4). Consequently, biodiversity leads to a lower risk of vector-borne diseases.

### Insects as tools

Blowflies (Calliphoridae), and their maggots help in forensic examinations to determine time of death, site of crime, even drugs ingested by the deceased [50]. Maggots of the greenbottle blowfly (*Lucilia sericata*) assist in the debridement of non-healing wounds of soft tissue and bones. Used for thousands of years in various cultures, and their benefit in the treatment of wounds recorded since the 16^th^ century, William Baer (1872–1931) employed maggots with great success where antiseptics did not work [51]. The arrival of sulphonamides and penicillin at the same time promised success without repulsion, and maggot therapy met with contempt and ridicule in the 1980s [52]. Ironically, with multidrug-resistance, modern ‘biosurgery’ has been enjoying a renaissance since the 1990s [53, 54].

Insects support scientists in many ways. The fruit fly *Drosophila melanogaster* is not only familiar to high school students discovering genetics, but is crucial in biomedical science [55], including cancer studies [56]. Insects inspire novel ideas, for example, in architecture [57] or assist in research in biomechanics, developmental biology, evolution, physiology, ecology, climate change and, long dead and in sediments, furnish paleolimnologists with information to reconstruct past environmental states of inland waterways and lakes [40].

### Cultural and economic role

Insects were revered religious icons, such as the sacred scarab in Egypt [40]. The honeybee is important in many cultures for food, medicine, art, and spirituality [58]. It features in artworks from as early as the Mesolithic period to Egypt, Greece, China, and Europe into the 21^st^ century as a symbol of industry, fertility, wealth, and power [41]. Bees may contribute to achieving 15 UN Sustainable Development Goals [59–61], a topic to which *Current Opinions in Insect Research* devotes an entire issue (2020, vol 40). A particularly inspiring example is insects’ contribution to peace in the shape of reintegrating Colombian ex-combatants as smallholder farmers to produce black soldier flies for livestock feed [62]. The rearing or collecting of insects for food, jewellery or scientific laboratories supports local women around the world. The presence of spectacular species, such as the migrating monarch butterfly, glow-worms in caves, or butterfly parks provides a basis for successful ecotourism enterprises and income for local people leading to a reduction of logging and pesticide use. The once thought extinct, and for some time ‘rarest insect in the world’, the Lord Howe Island Stick Insect (*Dryococelus australis*), attracts through its story alone and awaits reintroduction after the expected extermination of the black rat population on Lord Howe Island [63].

Insects play a crucial role in mitigating a long list of global anthropogenic challenges [44]. With poor image and met with indifference or dismay, the demise of insect species does not alarm most people. More funds go into controlling pests and vectors than into conservation. Yet, for humans to continue enjoying insects’ vital services, the call is for ‘a strong focus on preservation and conservation of as many, and as large as possible, pristine and near-pristine unique and typical landscapes as soon as possible’ ([64], p.258). Comprehensive recent alerts to the worldwide decline of insects suggest the need for urgent and purposeful action to combat the main drivers [30, 65, 66].

## Insects causing harm

Not all insects serve us well. While all have their place in the complex and balanced ecosystem, deforestation [67], land use changes [68], favourable breeding conditions provided by rains after drought, wind and temperature, and an abundance of food, e.g., monocultures, grain stores and new food sources, such as lawns, furniture and timber houses, can turn insects into pests. Beetles (Coleoptera – the largest insect order) represent a wide variety of destructive species. Cane beetles devastate sugarcane plantations. Borers, supposed to eat diseased and dead wood, attack living trees, and furniture. Weevils destroy grains. Beetle grubs devour roots of lawns and ornamental garden plants; caterpillars eat the leaves. Of the over 3000 termite species (Isoptera), beneficial in fertilising and improving soil, fewer than 30 are invasive and attack human-made structures [69]. Since antiquity, swarming grasshoppers (Orthoptera), and especially locusts, made their name by obliterating entire crops, often leading to famine. Others, such as cockroaches (Blattodia), ants (Hymenoptera) and house/stable/latrine flies (Muscidae/Fanniidae) impact on human health by transferring pathogens to food, food-contact surfaces and water [70, 71] or triggering allergic reactions [72]. The decided enemies of human health, however, are those arthropods that pierce human skin and transmit viruses, bacteria and parasites.

## Medical entomology

While entomology is the study of insects, medical entomology includes other arthropods, the Arachnida, such as ticks and mites, if they play a role in human health. The latter will only be mentioned in passing but the central theme of this paper extends to them. Even without transmitting disease to humans, pets and livestock, many insects, especially mosquitoes, are a nuisance [73] due to their buzzing insistence in contact, afflicting itching bites, sleepless nights, and possible superinfection after scratching. This problem is worst in places of known seasonal abundance, such as arctic and subarctic regions [74, 75], making life outdoors intolerable. Of graver concern are those insects that transmit serious illness. We know of the prehistoric origin of insects through palaeoentomology, while the prehistoric origin of pathogens is the realm of palaeomicrobiology [76]. Ancient microbial DNA extracted from fossil ice, rock salts, amber, bones, dental pulp or coprolites sheds light on the evolution of microbes to ancient diet, and health and disease [77], including ancient key pathogens causing plague, tuberculosis, leprosy and cholera [78]. Palaeovirology studies ancient viruses, and how ancestral hominids may have survived infections [79]. Palaeoparasitology examines fossilised evidence of parasitic stages [80] and the early forms of ancient hosts, evolution and distribution via ancestral insects of paleoplasmodia [81, 82] or the two palaeoleishmania in 100 million years old and 20–30 million years old amber [83–85]. Despite modern molecular genetic analysis, where ancestral pathogens came from and how they entered and co-evolved with vectors is the topic of several yet unconfirmed theories [84].

The breadth of arthropod-borne disease transmission can only be summarised here as a reminder. Medical entomology reference texts provide the necessary details. Mosquitoes (Culicidae) of interest to human health are divided into Anophelinae and Culicinae. Various *Anopheles* species transmit parasites causing malaria and filariasis. Mosquitoes of the genus *Culex* and *Aedes* are the most important vectors of yellow fever, dengue, Japanese encephalitis, chikungunya, zika and various other viruses and filarial parasites. Blackflies (Simuliidae) transmit a parasite causing onchocerciasis (river blindness). Sandflies (Phlebotominae) spread various forms of leishmania parasites, bacteria causing bartonellosis and viral sandfly fever (Pappadaci Fever), while biting midges (Ceratopogonidae), apart from being an extraordinary nuisance, transmit filarial and viral disease. Horseflies (Tabanidae) cause painful bites and spread the nematode *Loa loa*. Tsetse-flies (Glossinidae) are important parasite vectors of African trypanosomiasis (sleeping sickness). The larvae of some flies, such as botflies (Oestridae), fleshflies (Sarcophagidae) and blowflies (Calliphoridae), cause great psychological and physical bother by invading subcutaneous tissue on any part of the human body. Fleas (Siphonaptera) present as biting nuisance but also transmit bubonic plague, typhus, tapeworms or, as *Tunga penetrans*, invade skin between toes or under toenails to grow to pea-size before releasing thousands of eggs. Of the sucking lice (Anoplura), the body louse (*Pediculus humanus*) may cause dermatitis and impetigo but is of concern as transmitter of louse-borne typhus and relapsing fever. Bedbugs (Cimicidae) can cause severe reactions to bites and iron deficiency in the vulnerable. Kissing bugs (Triatominae) transmit a parasite that causes Chagas disease if their infected faeces enters the bite wound. Finally, a few small Arachnida also transmit pathogens. Soft ticks (Argaridae) are predominantly responsible for tick-borne relapsing fever while hard ticks (Ixodidae) cause tick paralysis and a long list of viral and bacterial infections, such as haemorrhagic fevers, spotted fever, Q fever, Ehrlichiosis, Lyme disease and tularaemia. Scabies mites (Sarcoptidae) and scrub typhus mites (Thrombiculodae) conclude this general overview. What unifies all these vectors is the perforation of skin through which pathogens enter. No perforation – no illness.

## Fighting the miscreants

Considering the many different types of insects and their potential to carry so many diseases, while often sharing the same location, ‘shooting the messenger’ is the obvious strategy. As it turns out, not all insects are the same. To develop a successful strategy for control, their behaviour must be considered as their preferences regarding movement, oviposition, feeding and resting vary widely. This is certainly one reason why vector control so often fails. Some insects barely fly 100 m from their breeding grounds while others can fly up to 100 km; wind carries some a few kilometres, others over 500 km. Still others just clamber from one host to another or drop down from a branch. They lay eggs on water surfaces, above the waterline or on damp substrate. Others prefer flowing water or partially submerged items. Some insects require dry sand or soil while others prefer cracks and crevices in walls and ceilings. Some lay eggs into wounds, others onto clothing fibres, and some deposit not eggs, but ready-made larvae.

The medical interest lies in insects’ feeding habits, especially if they are exclusively anthropophagic, and endophagic (in-house) or exophagic (outdoors). Of many flying vectors, only the female feeds on blood, at least from the second batch of eggs, while in others, both sexes are blood-feeders. Some bite during the day, others at night; some prefer bright sunlight others feed just before dawn. The mouthpieces of some enable the biting through clothing. Insects’ resting places are also important for vector control, especially for mosquitoes, which may be endophilic (in-house) or exophilic (outdoors). Others prefer dark humid locations but dry surfaces, or specific vegetation, from ground level to various heights. In addition, what attracts insects to humans has been of burning interest for a very long time and will be discussed later.

## From pest control to vector control

From antiquity, humans had to deal with pests in many forms, predominantly to protect food stuff from rodents or a myriad of insects [86, 87] or to escape the inconvenience caused by mosquitos, flies, lice, bedbugs and others. Typical strategies included magic and cultural practices, predators such as cats or pythons, but also the application or fumigation with chemical and organic substances, such as camphor, sulphur, copper, aromatic plants, fragrant wood or spices, possibly based on trial-and-error [88]. The *Papyrus Ebers* [89] recommends date-meal against fleas and lice, and fascinating historical reviews suggest innumerable and puzzling strategies to rid of a vast assortment of pests. The extraordinary efforts in fighting bedbugs (*Cimex lectularis*) through history, incredibly still a problem today, are presented in two delightful accounts [90, 91].

While old scripts and artefacts suggest that there was some awareness of a link between some pests and ill-health, only the modern understanding of life cycles, pathogens and transmission of disease turned pest control into vector control. Today, a range of methods is employed together with techniques from other disciplines, e.g., Geographic Information Systems [92] and mathematical modelling [93] as part of integrated mosquito management programs.

## Vector control

An enormous amount of literature reflects the evolution of control methods depending on the then knowledge of insects’ life cycles and behaviour, and the available technology. Because of the large variety of species, and most research focusing on mosquito control, the methods listed here [94] apply primarily to mosquitoes (and flies) as the main example unless otherwise indicated. Unsurprisingly, this discussion is heavily summarised but highlights advantages and disadvantages of each method.

### Physical control

Physical control measures interrupt insects’ life cycle and target either larvae or adults.

#### Immature mosquitos

Oils, surface films and polystyrene beads block mosquito larvae’s breathing. These measures also prevent oviposition on water.

##### Petroleum oils

In ancient Japan, whale oil on water surfaces dealt with undesirable insects [22]. William Gorgas applied his Cuban experience by using petroleum oil as one of the many measures to control the mosquitos responsible for the high death toll to yellow fever and malaria during the construction of the Panama Canal [95]. More paraffinic oils block larvae’s siphons necessary for breathing (more aromatic oils are larvicides, i.e., not physical measures). However, oils affect non-target aquatic and surface life, and may degrade through bacterial colonisation.

##### Surface films

These modify the air-water interface by reducing the water surface-tension, and larvae drown. Some are usable on potable water, but all affect non-target aquatic and surface life.

##### Polystyrene beads

The water is covered with non-biodegradable expanded polystyrene beads (2-5 mm) spread over confined breeding sites. Beads have been used with some success in various countries, especially in pit latrines and water tanks. Unless on safely and strictly confined water surfaces, the use of beads does not sit comfortably with our current understanding of plastics and microplastics escaping into waterways and oceans.

#### Adult mosquitos

##### Mass trapping

Commercial gadgets trap insects with light, CO_2_, heat, or attractants. Their usefulness on a large scale is questionable. They trap non-target insects, although a male *Aedes aegypti*-specific sound trap was recently developed [96].

##### Screens

Window and door-screens provide a barrier if properly maintained to enjoy an insect-free living and working space. Convenient face nets, attached to a hat, prevent nuisance landings.

##### Bednets

Plain bednets work similarly if positioned correctly. However, they offer hiding places for mosquitos in the folds when rolled up during the day. Insects are then often locked in, instead of locked out. Smaller insects, such as Phlebotominae, can get through conventional mosquito nets, and denser nets are required, which are, unfortunately, too hot, and unpleasant.

##### Other methods

Attempts to burst mosquitos with laser or high-energy ultrasound devices are not yet developed satisfactorily and affect non-target insects.

The advantage of physical control is the improbability of resistance. While there may be positive results in specific locations, physical control is of limited use in larger areas and in natural environments, and mosquitos eventually return.

### Environmental control

Before the arrival of synthetic insecticides, environmental control measures were the main technique against mosquitos. These techniques are back in favour with the increasing resistance in the target population to chemical control. Modifications to dwellings in recognition of local mosquito behaviour achieve positive results. However, the principle of environmental control is the removal of oviposition sites, mainly water. In urban areas, this means standing water in containers, especially ‘productive’ containers [97], in and around houses (empty bottles, food trays, toys, gadgets, tyres, flower vases), gardens (palm fronds, bromeliads, coconut shells, rubbish, discards), construction sites, cemeteries, rubbish tips, blocked drains, sewage and waste-water processing, but also design-faults, such as uneven surfaces prone to pooling. The importation of vehicle tyres, especially unwrapped, i.e., smuggled, from endemic countries requires relentless surveillance [98] as does other passive dispersal [99]. Unknown or unreachable water bodies hamper any efforts. The most prominent agricultural breeding sites, rice fields and large flooded areas after prolonged rains, benefit from intermittent flushing or draining/filling in, but the results are not overly satisfactory and may attract other species. Water in the natural environment, be it in leaf-axles, animal footprints, or extensive wetlands, is impossible to remove. Drainage of swamps and marshes has made way for newer techniques that protect local wildlife and biodiversity including natural vector predators. Environmental control relies heavily on community education [100], acceptance, involvement, and ownership.

### Chemical control

Continuing from the historic use of chemical elements and then arsenicals, the first synthetic organic insecticides appeared at the end of the 19^th^ century. In the early 20^th^ century, the chemical industry developed progressively stronger compounds, producible in large quantities and applicable over large areas. In 1939, Paul Müller synthesised the chlorinated hydrocarbon dichlorodiphenyltrichloroethane (DDT), the astonishing attributes of which promised unrivalled success [101]. Other chemical groups followed: organophosphates, carbamates and, by the 1970s, pyrethroids. Unfortunately, apart from rapidly increasing resistance in target species, the immense immediate and long-term collateral damage to non-target species, natural predators, water, soil, air, plants, wildlife, human and animal health, and their intricate ecological links through the indiscriminate and excessive use, especially of DDT, against all evidence was alarming. Sixty years after its publication, now is a good time to re-read *Silent Spring* [102], one of the most influential books of the 20th century. It changed our approach to blanket pesticide use and undermined trust in governments and industry whose drive behind mass chemical treatment seemed unimpeded regardless of the consequences. The Stockholm Convention on Persistent Organic Pollutants of 2004 (revised in 2019) aims to reduce and eventually cease DDT use apart from mindful application in vector control [103]. The WHO lists DDT for indoor residential spraying against *Anopheles* [104]. Today, increasing evidence suggests that DDT is probably carcinogenic [105] and, alarmingly, causes transgenerational harm to descendants of those exposed decades ago, such as developmental, reproductive, and neurological abnormalities [106] as well as risk factors for breast cancer and cardiometabolic disease [107, 108]. Such evidence questions the ethics of WHO’s approved DDT use.

Globally, the use of DDT has slightly decreased but resistance to alternatives forced some countries (China, India, DPR Korea) to continue production and use and, especially India and southern Africa, against malaria and leishmaniasis. However, rising resistance against DDT and alternatives paints a bleak future for a large sector of conventional vector management strategies [109]. To replace DDT, newer insecticides with completely new modes of action, e.g., neonicotinoids (now found in bees and honey [110], and human urine [111]) have been developed. Insecticides require frequent reapplication. The ecological impact on terrestrial and aquatic ecosystems and communities through primary and secondary poisoning and sublethal effects [112] still applies. The worldwide pesticide (including insecticides) use has been estimated between 3.5 million tonnes for 2020 [113] and over 4 million tonnes for 2018 [114], half of the latter in China, followed by the US and Brazil [114]. Insecticides are applied as liquids, concentrates, powders, granules, sands, pellets, briquettes, or slow-release formulations. Aerosol sprays are used in households and aircraft disinsection [115, 116]; the section on personal protection will mention further applications.

### Biological control

Biological control has been employed for many centuries. In China, ant nests were sold to control pests [22]; in 3^rd^ century Yemen, ants controlled citrus insect pests [117]. To control locusts, Indian myna birds were imported in 1762 to Mauritius, spreading all over the world, and entering Australia in the late 19^th^ century where they are now a severe invasive pest. Since then, there were innumerable attempts of biological control, successful or not, until cheap chemicals arrived after WWII [117]. However, as discussed above, synthetic insecticides’ impact on ecosystems and human health led to an increased focus on less harmful alternatives. The typical biological control employs the inoculation or inundation of pest habitat with predatory fauna. Following the successful control in 1888 of the cottony cushion scale in California, biological control has been applied all over the world. However, without accurate knowledge of a predator’s biology (see the fateful importation to Australia in the 1930s of the poisonous cane-toad *Rhinella marina* against the sugar-cane beetle), there is a substantial risk of ecological damage as well as damage to native and non-target fauna and flora. Was the focus first on benefits, from the 1990s, the focus shifted to the risks of biological control. A balance between benefits and risks should guide biological control [118] with the overall aim to protect native predators. Today, several methods are available.

#### Predators

Larvivorous small fish (*Gambusia affinis* – attack also non-target species) are introduced to water bodies. Natural predators include amphibians, newts, insect nymphs, flatworm, hydra, water bugs, water beetles, carnivorous mosquito larvae, aquatic mites and spiders, and crustaceans (*Mesocyclops* spp. [119]). Adult mosquitos fall prey to birds, bats, beetles, insects, frogs, geckos and lizards, mites, and spiders. Some spiders are specialised mosquito predators [120]; some target blood-carrying females using the red colour as a prey cue [121]. The impact of the loss of natural predators on human health is evident in the massive decline in frogs and subsequent increase in malaria in Central America [122].

#### Parasites

Aquatic nematodes regulate mosquito larvae in their natural habitat but are not used as control tool. Parasitic insects play no role in commercial control yet.

#### Pathogens

Naturally occurring entomopathogenic fungi [123], protozoa (microsporidia), bacteria and viruses destroy larvae and adults. While viruses, so far, have proved unsuitable, exiting and constantly evolving research concentrates on the employment of bacteria. For example, the toxin of *Bacillus thuringiensis israelensis* is highly larvicidal against mosquito and blackfly larvae and, because of its complex action, less likely creates resistance. Male *Aedes* mosquitos infected with *Wolbachia pipientis* and released into the wild interrupt the reproduction of vector populations. The current focus is on dengue fever control but applies to other infections transmitted by *Aedes* spp., such as zika, chikungunya, yellow fever, many other arboviral infections and filariasis. Unfortunately, contrary to popular belief, male mosquitos (modified or not) are also attracted to humans. They do not bite but swarm [124] and, as a nuisance, get killed [125]. In some settings, the combinations of predation and fungal infections against *Anopheles* spp. showed promise [48].

#### Plants

Many plants contain compounds that protect them from predation. These same substances have been under investigation for some time as to their usefulness in biological control. Essential oils and other plant extracts have been tested as bio-pesticides in agriculture and horticulture as well as vector control options. Most have low dermal or neural toxicity in mammals. There is no bioaccumulation or magnification as with synthetic insecticides, but there are possible unintended effects on non-target animals [126]. In the past, the use of plant essential oils has faced several practical challenges and high costs compared to synthetic insecticides, but new technologies should overcome these barriers [127]. Today, the list of plants under investigation is too vast to name but includes chrysanthemum (pyrethrum), neem tree seeds, citronella, red mangrove [128], geranium, lemon eucalyptus, cedar, belladonna, cinnamon [129], ginger, soybean, lavender, mint, and basil [94, 126, 127, 130]. Utilising invasive weeds showed promise in Vietnam [131]. Like other plant alkaloids, such as pyrethrum or nicotine, the cocaine in coca leaves (*Erythroxylon* spp.) functions as a natural insecticide [132]. Similarly, plague locusts tend to avoid Qat bushes (*Catha edulis*) (pers. observation); extracts may have potential for affordable control strategies.

#### Diatomaceous earths (DEs)

These fossilised remains of phytoplankton show great promise in crop pest and vector control. They consist of the silicon rich external skeletons of unicellular algae. The dust desiccates, and its sharp edges possibly injure insects – including non-target species. It works alone or in combination with other control agents. DEs have been used to control bedbugs, kissing bugs, *Ae. aegypti*, and veterinary vectors [133].

### Genetic control

In the 1950s, the idea of genetically suppressing undesirable insect populations, then successfully employed against the New World screw fly, gained momentum thanks to the evolving recombinant DNA-technology and the sequencing of mosquito genomes. Genetic control can be seen as an extension to biological control where insects breed themselves out of existence. Today, there are two main strategies: population elimination and population replacement. The first relies on various techniques to release sterilised male mosquitos, whereas in the second, modified vectors that cannot carry a particular disease progressively replace vector populations. The benefits are that mosquitos disperse naturally and unrestrictedly beyond areas accessible to conventional methods. The insect becomes the control, thereby avoiding non-target impacts. As with all genetic techniques, there is the risk of unplanned consequences. Issues of legal and ethical concerns as well as community acceptance need addressing.

Each currently employed strategy has shortcomings (Table 2). To counteract these drawbacks, any alternative measures require close examination for potentially negative impacts.

**Table 2:** Main shortcomings of current vector control

**General**	• Impossible to rid the planet of every member of the target population • Impossible to prevent re-population of ‘cleared ‘area by other species with the potential to ‘learn’ and take over previous vector’s function • Destruction of other species, including predators • Development of resistance • Constant monitoring, resistance monitoring required • Damage control required • Breaks down during adverse events, such as pandemic, war, civil unrest, economic crises, natural disasters • Community participation needed • There may be different vectors in one area but only one is targeted • Impermanent success, unsustainable
**Physical control**	• Impact on aquatic and surface non-target aquatic and surface species • Environmentally questionable, e.g., plastic beads; microplastics in waterways • Surface oil cover degradation by bacteria • Maintenance of barriers required • Limited to no use in larger areas or natural habitat
**Environmental control**	• Potential replacement by other vectors • Natural habitat impossible to remove • Oviposition sites impossible to remove entirely
**Chemical control**	• Temporary; requires constant re-application • Resistance requires increasingly higher dosages and more frequent applications • Severe impact on non-target species • Severe impact on water, soil, air, human and animal health
**Biological control**	• Potential impact on non-target species • Environmental safety (e.g., toxins) • Potential resistance • Invasion of ‘control/predator’-species; may need chemical control -> return of original pest • Introduced species eat the ‘wrong thing’ • Locally restricted
**Genetic control**	• Potential unplanned consequences • Difficult to demonstrate effect • Expensive • Legal and ethical concerns • Absence of community participation

## Personal protection

While vector control targets insects, personal protection measures shield humans from insect bites by physical, chemical, and biological means, such as impregnated material, or spatial and topical repellents. Some work community-wide, others as personal protection by individuals as needed.

### Impregnated material

A mixture of physical and chemical control, impregnated nets and fabrics are to protect people from approaching insects.

#### Insecticide-treated bednets (ITNs)

The unsatisfactory performance of plain bednets led to pyrethroid-impregnated nets. Despite some positive results, e.g., against malaria [134] or Chagas disease [135], variations in protective efficacy [136] but also practical issues, such as ill-fitting and broken nets, unreliable dosage, the need to re-treat after washing, poor net-quality, progressive resistance to pyrethroids, and cost hampered the nets’ usefulness.

#### Long-lasting insecticide treated betnets (LLINs)

Circumventing some of those problems, LLINs undergo a single treatment at manufacture to be active for their entire life. A mainstay in malaria control, innumerable studies have tested acceptance and efficacy worldwide. A high reported (rather than observed) use of LLINs in Kenya showed significant protection against malaria [137]. Considering the serious consequences of malaria during pregnancy, protection is crucial. However, distribution and use proved unequal in Kenya, where more educated, affluent, and urban women had access to nets compared to their rural counterparts [138]. In Ghana, lack of knowledge, heat, lack of hanging facilities and no spousal support meant low use [139]. Free distribution to the poor often raises the question of reselling for items of perceived better value [140]. However, in the DC Congo, free distribution to pregnant women in a poor area in the capital achieved high use [141]. As with all nets, they are only useful during bedtime, not the biting time of respective insects. As ever, resistance to the insecticide needs addressing, though a simple field-test appears useful for early detection [142]. All public health initiatives are vulnerable to interruptions, including pandemics [143], causing setbacks or negating achieved success entirely. Recently described potentially decreasing bio-efficacy of unused LLINs may trigger rises in infections, and nets should be retested pre-delivery [144].

#### Other items

In Venezuela, the community preferred pyrethroid-impregnated curtains in Chagas control as more practical than bednets. In one study, they led to 100% sylvatic triatomine mortality [145]. Permethrin-treated uniforms, underwear, socks, and hats of Colombian soldiers protected from malaria and leishmaniasis [146]. In Cambodia, impregnated hammocks acted as additional protection of outdoor workers and villagers from malaria before bedtime [147]. Deltamethrin (ZeroFly®) incorporated in plastic sheeting, similarly to storage bags against infestations of grains [148], showed success in malaria control in displaced and mobile populations in highly endemic areas in India where traditional measures were impractical or impossible [149, 150]. Indoor use of impregnated sheeting may address pyrethroid-resistance [151] and surpass indoor spraying [152].

### Spatial repellents

Spatial repellents fill the air with vaporised insecticides, are available in a range of applications and protect more than one person at once. Mosquito coils, a Japanese invention from the late 1800s [88], consist of insecticide plus a combustible compound. They only need a match, are affordable and much used in developing countries. On ignition, the insecticide in the smoke repels mosquitos from approaching or entering rooms. It reduces bites but not necessarily disease. Some coils are only for outdoor use, some disperse disagreeable smells. The immediate and long-term impact of smoke pollution on respiratory health may be considerable [153].

Electric devices depend on a power source and require the purchase of compound replacements. They are available as vaporising mats or liquid vaporisers where heat (without smoke) releases insecticide vapour. Passive emanators, some with additional battery-powered fans [154, 155], require no electricity; ambient temperatures release insecticides. The alleged reduction of human-mosquito contact through coils and emanators requires more research with firmer methodologies [156].

In households, insect sprays serve as knockdowns or extended surface treatments; citronella or sandalwood candles, repeller lanterns, electrical zappers and a range of questionable gadgets provide limited to no effect. Though popular, there is no evidence that electronic mosquito repellents work [157] – but increase biting rates [158], and neither do ultrasound devices [159] or smartphones with ‘mosquito-away technology’ [160]. Again, aerial concentration of insecticides or other compounds may affect respiratory health. Research into botanical spatial repellents seems promising [161]. Plant-based fumigants may also be cheaper and culturally more acceptable [162]. All methods are flawed (Table 3).

**Table 3:** Main shortcomings of impregnated material and spatial repellents

**Impregnated Material**	Bednets • Problems with damage, poor fit, incorrect use, infrequent use, re-drenching • Loss of bio-efficacy • Lack of understanding, lack of community participation • They may not coincide with the insects’ activity cycle Fabrics/plastics • Spray on for short-term visitors’ clothing, not for locals • Potential skin irritations
**Spatial repellents**	Non-electric, electric, emanators, sprays • Reduce bites but not disease • Impact on non-target species • Smell, smoke, irritant • Some require electricity • Cost of compound replacement • Respiratory impact (smoke, insecticide) Ultrasound and electronic devices, app-devices • No evidence of effect

### Personal repellents

Several measures attempt protection by blocking insects from landing on skin, such as spraying clothing (especially for travellers or outdoor workers), wearing wristbands or other items with questionable results [163] but, most importantly, topical repellents, which also offer protection before bedtime under nets.

## Topical repellents

The use of topical repellents possibly predates humans as some primate species rub millipedes and certain plants on their fur during heightened mosquito activity [88]. Historically, and in many cultures, mud, oils, plant infusions and repellent adornments kept biting arthropods away. This practice may have been forgotten in the Middle Ages where barbers picked fleas and lice off their customers. The desperate lack of topical repellents over the centuries in Britain meant ‘gaol fever’ or ‘spotted fever’ bothered all from paupers to royals [164]. The development of synthetic repellents started during World War II with the US military facing millions of duty-days-lost due to malaria and scrub typhus [88]. Eventually, in 1953, DEET was discovered.

### Synthetic repellents

Still the gold-standard today [165] against which new compounds are tested, DEET (N,N-diethyl-3-methylbenzamide) repels a wide range of disease-carrying insects. It is available in various concentrations (5%-100%) and comes in many formulations: spray, lotion, cream, roll-on and more. Towelettes ensure a more even distribution but produce waste. Soaps showed mixed success [166] and proved impractical [167]. Controlled release systems, such as microencapsulation, prolong the time between re-applications. While an excellent repellent, its smell, oily stickiness, skin and eye irritation, and plasticising effect, i.e., damaging spectacle frames to synthetic fabrics, make its use unpleasant. A long list of practical guidelines also includes concerns of potential toxicity [168]. Currently, DEET is not recommended for children under 6 months and pregnant women [169]. Picaridin and IR3535 provide very good protection without DEET’s unpleasantness, the latter having reportedly led to ill-advised use of sulphur from matchsticks, diluted turpentine, or pet flea-and-tick-collars [170].

### ‘Natural’ repellents

Rising public demand for reduced exposure to chemicals accelerated research into bio-sourced alternatives. In 1966, the defence-discharge of some arthropods against predators was thought ‘repellent’, and its potential use ‘to protect crops, forests, man and animals’ suggested ([171], p.413). However, research turned to plants and, for decades, essential oils of numerous species have been investigated, the most common citronella, lemon eucalyptus, neem oil and a long list of other aromatic plants [172, 173], most recently nootkatone (grapefruit and Alaskan yellow cedar) [174].

Although most work focuses on mosquitos, plant-based repellents are tested against Triatominae [175], Phlebotominae [176, 177], Argasidae and Ixodidae [178, 179] and other vectors [130]. Furthermore, local plant-based repellents may be cheaper, more available, and culturally acceptable and, subsequently, applied more sustainably by local populations in endemic regions [162, 175, 177, 180]. For example, the juice of the unripe fruit *Genipa americana* (Huito) has been used by Amazonian people as insect repellent. The juice stains the skin dark-blue. The local belief that the disease comes at night and cannot see darkened people finds support in its repellence against *Lutzomyia* [177] and insects in general (see Table 1). The production of colourless juice seems a logical step. However, just because a topical repellent is ‘natural’ does not mean it is non-toxic [181] or its production is eco-friendly [182]. The rapid progress in nanotechnology raises hopes for the development of effective and safe compounds [183].

### Comparisons between synthetic and natural repellents

The long search for the perfect topical repellent is evident in the innumerable studies comparing a wide range of compounds, concentrations and commercial products employing a wide range of methodologies against a wide range of vector species, often with DEET as the gold standard. Synthetic products are compared [184], various mixtures with DEET [162], commercial products with DEET [185–187], plant-based compounds with each other [188]. The results vary from disappointing to questionable to surprising (Victoria’s Secret Bombshell fragrance [187]) to positive. Notwithstanding standardised testing guidelines [189], the rapid technological advances require new methodological standards [173]. Extensive reviews of repellents exist for travel advice [188, 190], a necessity when only few travellers apply the recommended dose [191] or use repellent in malaria endemic regions [192, 193].

### Problems with topical repellents

This is all very well when plans are hatched, and strategies designed, in air-conditioned offices or laboratories. In 36 °C heat and 98% relative humidity for the best part of the year, products and advice prove useless. Practical problems include the abrasion of treated skin through clothing, vegetation [194] or luggage straps, evaporation, absorption wash-off (rain, sweat), wind and high temperature [195]. Even with acrobatic effort, complete skin coverage is doubtful. Simultaneous use of repellents and sunscreen raises concerns. In some studies, DEET reduced sun protection [196], in others it did not [197]. More importantly, DEET and sunscreen together act as permeation enhancers, especially when repellent is applied before sunscreen [198]. Picaridin appears a safer candidate, yet, much more research is needed. 2-in-1 products may cause overexposure to repellents because of re-application requirements for sun protection [198]. Repellents act within very few centimetres of application [198]; vapours do not extend to nearby non-users, on whom biting rates increase [167, 195].

While the properties of the ideal topical repellent are clear: prolonged efficacy against a wide variety of arthropods, non-irritating to skin, odourless or pleasant odour, no effect on clothing (staining, bleaching, weakening), no oily residue on skin and cannot be wiped, washed or sweated off, inert to commonly used plastics, chemically stable, economically viable for broad use, nontoxic and of sufficient duration of effect [199], and no entry into the bloodstream [169], we still seem far away from this ideal (Table 4).

**Table 4:** Main shortcomings of topical repellents

**General**	• Complete coverage of exposed skin is impossible • No ‘proxy’-protection for nearby untreated skin or untreated persons • Practical problems, e.g., abrasion, wash-off, etc • Problem with simultaneous application of sunscreen • Complete coverage of a community with repellent is unrealistic • Reasons for non-use: ◦ tourists: sunscreen, long sleeves, hot and humid, sweat ◦ locals: unaffordable, unavailable, spoilage/deterioration, lack of storage facilities • Current and future resistance to various technologies in wild mosquito populations • Lack of user compliance in adequate and timeless reapplication • Low residual character of current repellent technology • Not always working • ‘Natural’ repellents may still be toxic or their production environmentally questionable
**DEET**	• Unpleasant, oily, sticky • Plasticises plastics and synthetic fabrics • Guidelines for use [195]; not use under clothing, clean palms (esp. children) after application to avoid contact with eyes, mouth, or mucosa; after exposure, wash treated areas with soap and water; avoid inhaling aerosol; avoid spraying into eyes (own and others), especially contact-lens wearers • Not recommended for children under 6 months and pregnant women

## Barriers to community acceptance of control measures

A wide range of control measures, used individually or in combination, form various vector control or mosquito management programs around the world with the aim to kill disease-carrying insects. Most strategies require community participation and ownership to be sustainably successful [119, 200]. Such acceptance relies not only on educational programs but depends on people’s changing circumstances, e.g., program fatigue, lack of funds, diminishing input from authorities and unforeseen interruptive events [100]. In some emergencies, a vertically structured technocratic and centralised approach may be successful in the short-term, but this is less sustainable and invites reliance on authorities [201]. On the other hand, increasing internet access can make innovative strategies acceptable, such as drone use in mosquito surveillance [202].

**Fig. 1:**
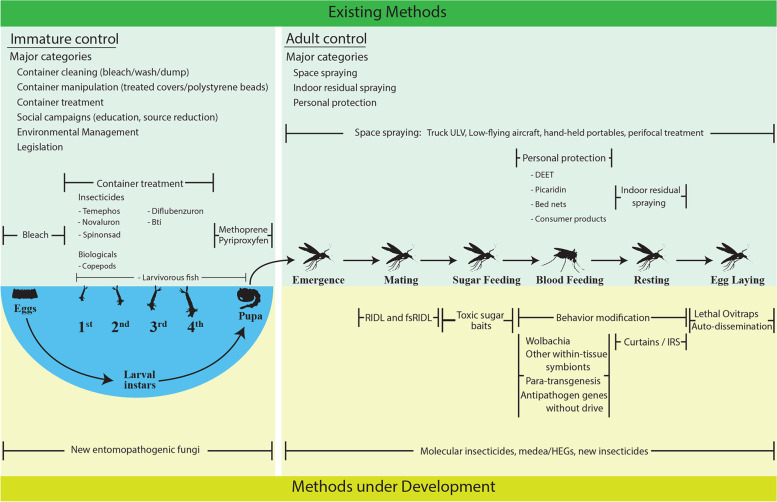
Overview of a multi-pronged approach in the example of dengue fever control

When the onus is on the individual to implement strategies, such as topical repellents, structural factors and socio-economic inequality influence the outcome. Poverty, lack of education programs and cultural understanding of illness hamper authorities’ desk-derived health directives around the world [138, 139, 203]. Using dengue control as example, Fig. 1 presents a comprehensive vector control plan with current methods and those in development [204]. Community involvement could be added.

Despite the plethora of vector control and personal protection measures, doubts have been raised not only regarding resistance [205], but the overall lack of reliable evidence for effectiveness of control measures [206] or their disease prevention [204], yet, proposals for remedy include combinations of current, deemed unsatisfactory, approaches [207]. So far, things do not look so great. Considering all previously discussed shortcomings, this should not surprise but rather lead to the question: is there anything that can prevent insects approaching us in the first place?

## What makes humans attractive?

The quest of finding what makes us so irresistible to certain insects has been the focus of long-standing speculations and enthusiastic experiments. ‘Sweet’ blood, the level of cleanliness, eating bananas, cheese and smelly feet are among many suspects. In the 19^th^ century, the sound of dynamo brushes or persistent ‘whooping and humming’ attracted male mosquitos to the surprise of the experimenter who suggested using a tuning fork to find the exact musical note the insects liked [208]. Elsewhere, mosquitos disliked octogenarians, psoriatic skin, and dry skin [209], while they completely disliked caprylic acid (goat odour) and could not find a human host rendered odourless with bleach [210]. Multiple studies led to skin factors, such as carbon dioxide, temperature, moisture, odour, and warm-moist convection currents as well as skin colour, sex and age [211] and lactic acid [212]. DEET, in the absence of lactic acid, appeared to attract insects when offered without a host, suggesting that DEET inhibits the attraction to lactic acid rather than being a true repellent [213]. Modern research into vector sensory physiology, building on entomologist Dethier’s ground-breaking research into chemoreception in the 1930s [214], demonstrates a complex spatial and temporal web of olfactory, visual, mechanical, acoustic, hygric and thermal signals [215]. *Plasmodium* infections render human odour attractive, and host olfactory cues can be learned and linked to a target’s likely defensive behaviour [215]. Such insights make the assumption that garlic and others attract or repel a clumsy proposition. In reductionist fashion, the search for the one attractant looks futile when synergy is at play. At the time of writing, a surprising discovery may allow a re-direction of research efforts that have, so far, largely been disappointing. Rather, as expected, each odour neuron expressing one specific odour receptor, individual odour neurons carried multiple different receptors making the mosquito olfactory system practically fail-safe [216]. An unexpected barrier for researchers, this discovery nevertheless opens a fresh approach to finding the elusive human-mosquito attraction. Given that so much is at stake, a strong focus on research into attractant physiology is required, paving the way for an entirely new, perhaps more challenging, research direction.

## The oral repellent – early disappointment

Another way of protection would be turning attractive hosts repulsive and so safe from disease. Research into attraction raised the question: what if we could eat something that keeps insects away? For almost a hundred years, the desire grew for a systemic repellent, but seemed a futile proposition [168, 195, 199, 210, 211, 217–220]. Nevertheless, South African research in the 1930s led the way. A solid dose of brimstone (sulphur) and treacle excreted in local workmen’s sweat displeased mosquitos; ‘what does it matter to an employer of labour if the workmen do smell of sulphur!’ ([221], p.594). The results were confirmed [222], though the term ‘prophylactic’ is misplaced. A further confirmation expressed hope that it should be possible to find an oral substance to neutralise body odour [210]. In the 1960s, US defence forces supported much research to combat the many deployment-days lost to vector-borne diseases. Published work concentrated on attraction and potential study designs [209, 218, 223] but no results seem ever obtained and available, even on request, suggesting that the idea came to nothing and was abandoned, possibly when topical repellents seemed more promising. Others continued with zeal to hunt the illusive element by feeding or injecting innumerable substances from repellents, hormones, coffee, deodorants, odoriferous fruit and many more [217] to DEET-injections [224] or Griseofulvin [225], without success. Hospitalised patients taking 144 different medications did not repel mosquitos either [226]. The most persistent suggestion is the use of thiamine chloride (Vitamin B). Recommended in the 1940s and disproved since [220, 226–229], it is still recommended by some doctors [220]. Garlic has been discredited [219], unsurprisingly, since people with much garlic in their diet are still getting bitten. Fanciful oral repellents, marketed online, led to the warning by the US Food and Drug Administration that such over-the-counter drugs are subject to regulatory action, until there is clinical evidence [230].

From the 1930s, the search for a systemic compound to repel insects resulted in passionate but, with rudimentary knowledge of mosquito physiology and crude technology, uncoordinated one-off attempts which can easily be viewed today with some indulgent amusement. However, when Schreck asked for an entirely new concept of mode of action in 1977 [231], he could not have foreseen that, 50 years later, still no fresh approach is evident. All the while, since the 1960s, the wish list for the ideal systemic repellent included effectiveness against a wide variety of arthropods, oral route of administration, long duration of action (12 to 24 h or longer after ingestion), low toxicity, and long shelf-life [218] as well as a concentration just sufficient to prevent penetration of a host’s skin [223]. Today, this ideal needs expansion with attributes that counteract the shortcomings of all control measures highlighted earlier.

## Current oral vector control strategies

Current oral means to combat vector-borne diseases include all treatments that reduce the uptake by arthropods of agents in the blood (e.g., malaria); they do not repel. Ivermectin in mass distribution against lymphatic filariasis kills blood microfilaria, breaking the lifecycle. Even though the drug demonstrated incidental mosquitocidal or life-shortening effects [232, 233], it does not repel. Recently, neuropeptide N receptors’ potential for food intake regulation happened to also block mosquito bites [234], but taking medication unnecessarily cannot be a solution in the quest for repellents. No doubt, more such chance effects will be noticed, but chance cannot be a pathway to address global disease. In veterinary medicine, dog chews against fleas, ticks and heartworm (*Dirofilaria immitis*)-transmitting mosquitos require the vector to attach first to be killed by the compound [235], with the risk of transmitting disease during the bite. They do not repel, and the small risk of transmission is not acceptable either. Something is needed to prevent biting in the first place.

## Revisiting a futuristic dream

With rapidly evolving nanotechnology and new insights into mosquito physiology, especially chemo-sensation, finding the still elusive compound that meets the desired criteria should be the focus of future research. Once this is found, its administration seems less difficult. One possibility is a repellent pill suiting individual short-term application, e.g., for travellers, scientists, military personnel, missionaries, and others entering endemic regions for limited durations. Depending on the substance, it may need to be taken before entering the area and regularly during the stay. The individual is in control and responsible for behaviour change, and compliance. However, this strategy cannot apply to large at-risk populations. Mass-distribution of tablet-based treatment for lymphatic filariasis, for example, still must overcome finance, supply, infrastructure and compliance issues [236] and is vulnerable to adverse events, political instability or natural disasters. Something is needed where ‘the whole population benefits without the need for individuals to change their behaviour or comply with advice of health professionals’ ([237], p.6). Ideally, a compound is added to something people consume daily, and is automatically delivered, not dispensed. Fortunately, this strategy exists already: the fortification of universally available commodities. For example, the fortification of flour with folic acid, mandatory in 80 countries [238], has significantly reduced neural tube defects in children [239]. The biofortification of staple crops to combat micronutrient undernutrition with Vitamin A (cassava, maize, sweet potato, rice (‘Golden Rice’ [240, 241]), iron (beans, pearl millet) or zinc (rice, wheat) feeds those without access to sufficient fruit and vegetables [242]. However, preferred staples vary around the world and developing individual fortification methods is unfeasible. Fluoridated drinking water to reduce tooth decay where natural supply is low has been supplied since the 1940s [243]. However, water is an unsuitable carrier of a repellent since large regions of the world do not have access to a reliable continued supply of purified drinking water. That leaves one commodity that has proved a successful carrier for over 25 years: table salt.

## Repellent-fortified salt – the way forward

The overwhelming success of iodised salt programs, mandatory in 124 countries and used by 88% of the global population [244], to address iodine deficiency disorders, i.e., stunted growth and development in children, led worldwide to major improvements in health and economic returns [245]. Unlike other commodities, salt is consumed relatively consistently around the word [236]. It can be transported, widely distributed and stored easily. It can be obtained by the poor or cheaply supplied to them, particularly important in countries with chronic and severe undernutrition [244]. All countries have established salt production and distribution systems [236], facilitating fortification strategies.

The advantages of salt-fortification to benefit large at-risk populations are plentiful. There is already an iodised salt infrastructure with salt producers familiar with the techniques. Close partnership between health authorities and the industry, involving salt representatives early, has proven successful as has the strategy to distribute through alimentation rather than health channels, avoiding the impression of medicalisation. Governments’ responsibility is consumer education, monitoring, importation requirements and quality assurance among others [246]. For example, in Sarawak/Malaysia, only iodised salt may be sold [247]. Even better, iodised salt can piggyback other compounds. Diethylcarbamazine (DEC)-fortified salt to eliminate lymphatic filariasis may not only overcome some obstacles of tablet-based programmes [246, 248] and, in Haiti, led to cost-effective social enterprises [248], adding DEC to iodised salt is technically straightforward [246, 248]. Adding the yet elusive repellent to iodised salt would ensure a wide benefit to millions in at-risk areas whom current vector-control cannot protect (Table 5).

**Table 5:** Benefits of oral repellent and repellent-fortified salt mass-administration

**General Benefits of oral repellent**	• None of the shortcomings of current control measures • Not affected by sweating, washing, swimming, rain, skin condition • Works on any kind of skin • No problem with sunblock, abrasion, body lotion, dense sun-protective clothing, sweat under raingear, etc • Complete coverage of insect-accessible skin
**Benefit of community-wide administration via salt fortification**	• Independent of availability of pills, quality control, affordability, good condition, correct use • Replenished with salt purchase • No additional action required by consumer • Covers wide geographical areas • Covers large populations

## Discussion

Most vector-borne diseases occur in poor or tropical regions of the world, particularly in rural and geographically disadvantaged areas away from central government. Millions cannot afford not to work in swamps, paddies, clearings, logging, or live in houses or areas that harbour vectors. Despite all the technological development and scientific breakthroughs, people still suffer illness, social stigma or die because they are bitten by insects. Treating diseases population-wide clearly is not successful, otherwise, insects would not be able to take up infectious agents. A combination of current strategies may deliver reasonable protection for some time, but they still do not cancel out their shortcomings, and many places cannot afford this luxury in the first place. Control programs usually target one species. In regions with several vectors, entirely different approaches may be required. People lucky enough to have access due to their geographical situation, e.g., small islands or isolated valleys, their inclusion in field trials, or the political will of a leader, will be fortunate; others will miss out. Much logistic and financial effort needs to go into vector control and personal protection, making their sustainability questionable and highly vulnerable to adverse events. At this point, no affordable mass-protection exists. As unpalatable as it may be, especially for those investing in and conducting research to rid the world of vectors, vectors are still here, are here to stay, and ensure known and emerging outbreaks.

Mosquitos typically go through multiple egg-laying cycles; each time a bite puts the next human at higher risk. While killing insects seems obvious, it does not address the root cause: the piercing of skin, not dissimilar to the weight-loss industry with its never-ending ‘new’ pills, gadgets, diets, therapies, and nonsensical advice when, for most, the root cause is: food passing lips. Mosquitos are not the deadliest animal on earth to humans, as so often proclaimed. Mosquitos are not the villains; the infectious agents are. Insects are merely the unhappy, involuntary, and sick carriers, infected by unwanted stowaways, harmful to their existence. They are invaded by microfilaria, viruses, gametocytes, amastigotes, or bacilli, settling in muscles, penetrating stomach and gut walls, blocking proventriculi or infest salivary glands and, depending on the invader, are mere facilitators of the completion of life cycles. Therefore, the infectious agents need to be eradicated, not the vectors [249]. If vector species are wiped out, others may move in, adapt to new habitats, and take over vector-function [250], suggesting that pathogens co-evolve with other insects. Rather, the goal is: no infected arthropods.

One Health claims the interconnectedness between humans, animals, and the environment, but it tends to focus on the human benefits of this arrangement. Calls for crucial closer links between the three systems to combat NTDs stay in the realm of the currently known with predictable suggestions [251], but ultimately go around in circles. The proposed strategy meets Action Track 3 ‘controlling and eliminating endemic zoonotic, neglected tropical and vector-borne diseases’ of the recent *One Health Joint Plan of Action* (2022–2026) by the quadripartite FAO, WOAH, UNEP, and WHO [252] but goes further by putting healthy insects (animals) back into their respective habitat (environment) and place in the food chain.

The fortification of commodities with micronutrients or therapeutics has a long and successful history. The general availability of salt and the fact that everybody, including the poor, need to eat it, makes salt the perfect candidate for a bold approach to mass-protection from vector-borne diseases, once a substance is found that makes humans unattractive blood-donors. If a safe compound could be developed to attach to table salt, millions of people could be protected without the need to think about protection, which can be unaffordable or is forgotten. Eventually, this method could be adapted to carry substances repelling other animals’ attack on human skin, such as hookworms or ectoparasites, in the way vaccines evolved from skin scratches with substrate to gen technology. The administration of oral repellents could cease, once diseases are limited to very small pockets responsive to treatment and eradication. This approach may even become applicable to pets and livestock.

Oral repellents do not mean that insect species will die out. Some mosquitos do not need a blood meal for the first or all batches of eggs (autogeny) [94]. Only Anophelinae and Culicinae feed on blood but autogeny exists in the genera *Culex*, *Anopheles* and *Aedes*. *Ae. aegypti*, in the right conditions and of favourable genotype, was found to be autogenous [253]. Lacking access to blood, they become part of the ecosystem where they belong, in numbers sustained before habitat interferences created an abundance.

What failed decades ago due to lack of knowledge and inadequate technology has now a chance of success. Trapped in funding and expectations, current vector control research will continue, but it is highly questionable if the plethora of shortcomings will ever be addressed. A second pathway with equally solid funding into the intricacies of mosquito chemo-sensation could form the basis for developing suitable compounds for oral repellents to reach all those people in at-risk regions whom current vector control has long excluded. This requires multidisciplinary cooperation, working across fields, thinking outside the box, and allowing fresh ideas and unorthodox methods to bring originality to the topic. This task may seem more daunting than finding universally successful vector control – but there is no promising alternative on the horizon.

## Conclusion

This paper started with an invitation to reacquaint ourselves with insects whose value we underestimate grossly. Without them we would not be alive, but our vector control efforts kill them regardless – especially those that have no business in transmitting diseases. Millions of people around the world are affected by vector-borne diseases despite the enormous investment in vector control and personal protection. Most affected areas are resource poor. The question of how much effort would be put into this problem if the non-poor were affected at the same magnitude may well come up soon when mosquitos move into warming ‘temperate’ regions of the globe (see *Aedes albopictus* in France [254]).

Sixty years ago, the idea of an oral repellent seemed ambitious and impossible. Yet, the thought of running one’s life via smartphones would have been equally outlandish. Times have changed with leaps in scientific knowledge and rapidly evolving ground-breaking technology. Without other options, two roads are at our disposal: 1) continue the current path with laboratories working busily, and little appreciable deduction in preventable diseases, and 2) start a second pathway with fresh imaginative approaches. We are thinking of moving to Mars, but we cannot prevent tiny animals from piercing our skin. This paper may upset a great many people, especially those who put decades of hard work into vector control and repellents. However, killing all vectors is impossible, as is 100% effective personal protection. When the message (infectious agent) is unwelcome, there is a tendency to shoot the messenger (insects). We ought to be able to find something better, something that protects people and, at the same time, assists in mediating the current rapid decline in insect populations. The food chain is re-established, and insects, including natural predators, return to our benefit. We have already most of what is needed: technology, entrepreneurship, and possible means of distribution. The intrepid idea of oral repellents and repellent-fortified salt raises hope for populations excluded from vector control and medical treatment. Scientific courage and creativity are needed – billions of people in endemic regions depend on it.

## Data Availability

Not applicable.

## Abbreviations

FAO: Food and Agriculture Organisation
UNEP: United Nations Environment Program
WHO: World Health Organisation
WOAH: World Organisation for Animal Health
